# Identification of Human UDP-Glucuronosyltransferase 1A4 as the Major Isozyme Responsible for the Glucuronidation of 20(*S*)-Protopanaxadiol in Human Liver Microsomes

**DOI:** 10.3390/ijms17030205

**Published:** 2016-03-09

**Authors:** Jia Li, Chunyong He, Lianxiang Fang, Li Yang, Zhengtao Wang

**Affiliations:** 1 Department of Pharmacognosy, China Pharmaceutical University, Nanjing 210038, China; lisa02252@163.com (J.L.); 13764556163@126.com (C.H.); 2 The Ministry Of Education Key Laboratory for Standardization of Chinese Medicines and the SATCM Key Laboratory for New Resources and Quality Evaluation of Chinese Medicines, Institute of Chinese Materia Medica, Shanghai University of Traditional Chinese Medicine, Shanghai 201203, China; lianxiang1205@163.com; 3 Institute of Chinese Materia Medica, Shanghai University of Traditional Chinese Medicine, Shanghai 201210, China

**Keywords:** UGT1A4, liver microsomes, 20(*S*)-protopanaxadiol, glucuronidation, liquid chromatography-mass spectrometry (LC-MS)

## Abstract

20(*S*)-protopanaxadiol (PPD), one of the representative aglycones of ginsenosides, has a broad spectrum of pharmacological activities. Although phase I metabolism has been investigated extensively, information regarding phase II metabolism of this compound remains to be elucidated. Here, a glucuronidated metabolite of PPD in human liver microsomes (HLMs) and rat liver microsomes (RLMs) was unambiguously identified as PPD-3-*O*-β-d-glucuronide by nuclear magnetic resonance spectroscopy and high resolution mass spectrometry. The chemical inhibition and recombinant human UDP-Glucuronosyltransferase (UGT) isoforms assay showed that the PPD glucuronidation was mainly catalyzed by UGT1A4 in HLM, whereas UGT1A3 showed weak catalytic activity. In conclusion, PPD-3-*O*-β-d-glucuronide was first identified as the principal glucuronidation metabolite of PPD in HLMs, which was catalyzed by UGT1A4.

## 1. Introduction

Triterpenoid saponins exist widely in many medicinal plants including ginseng, America ginseng, and notoginseng. Many saponins like ginsenosides might be totally or partially hydrolyzed in gut after oral administration. As a result, the saponins are normally considered as the prodrugs and the aglycones may act as the real pharmacophores exhibiting related biological activities [1]. Therefore, the evaluation of metabolites identification and metabolic pathway of the aglycones would provide a feasible access to these bioactive triterpenoid saponins for further understanding and application.

20(*S*)-protopanaxadiol (PPD) is one of the representative aglycones of ginsenosides metabolized from numerous dammarane type saponins through stepwise deglycosylation catalyzed by gastric acid or gut organisms. It has been stated that PPD may be the actual executers of ginsenosides [1,2,3,4]. PPD itself also has a wide spectrum of pharmacological activities, such as anti-inflammatory [5], antidepressant [6] and anticancer [7,8,9]. PPD is one of the most potent anticancer agents among the ginsenosides proved by the structure-activity relationship study, which can induce Hep G2 cell apoptosis by the endoplasmic reticulum stress pathway [10,11].It is reported that PPD has been regarded as a potential antidepressant by early clinical trials [12].

Previous studies disclosed that oral bioavailability of PPD was determined to be approximate 30% [13], and it was mainly eliminated via oxidation of the double bond at Δ^(24,25)^ to yield 24,25-expoides, followed by hydrolysis and rearrangement to form the 24,25-vicinal diol and the 20,24-oxide derivatives [2,14]. Yet the information related to the phase II metabolism of PPD is very limited. Li, *et al.* [2] reported that direct glucuronidation of PPD only occurred in pooled human liver microsomes and human hepatocytes when it was transformed into 20,24-oxide form. In our previous study, a glucuronidation metabolite was detected in rat urine, bile and plasma [15]. However, the location of the glucuronic acidmoiety and the isozyme(s) catalyzing this reaction remain unknown. Glucuronidation has been increasingly recognized as an elimination and detoxification for xenobiotics and endogenous compounds, which accounts for more than 35% of all phase II drug metabolism, where the UDP-Glucuronosyltransferases (UGTs) play an important role [16]. Until now, information regarding the glucuronidation of PPD is still limited. Our present study aims at identifying glucuronidation metabolite of PPD and characterizing the UGT isozyme(s) catalyzing the metabolism in human liver microsomes (HLMs) and rat liver microsomes (RLMs), in order to assist the understanding of elimination of PPD as well as PPD-type ginsenosides.

## 2. Results

### 2.1. Identification and Structural Elucidation of Metabolite

**Figure 1:**
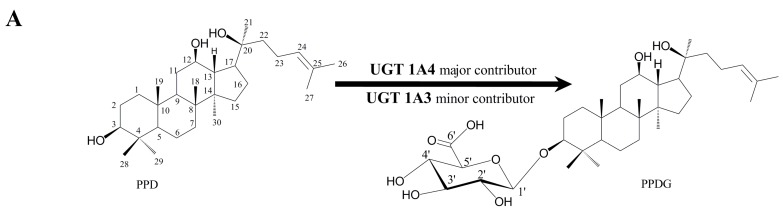

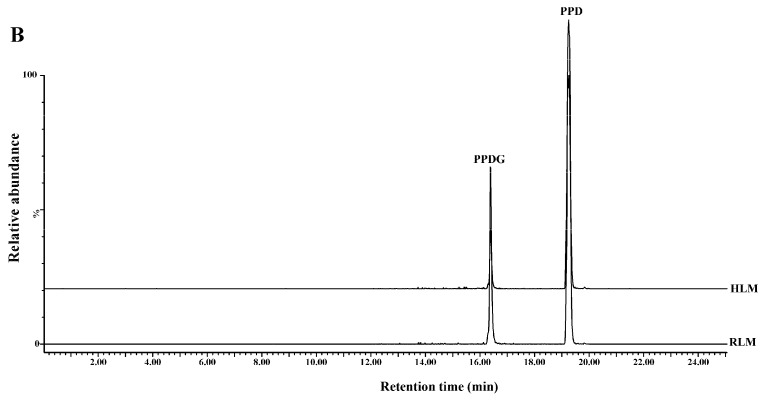
Chemical structures of 20(*S*)-protopanaxadiol (PPD) and its glucuronidation metabolite catalyzed by human UDP-Glucuronosyltransferase (UGT) 1A3 and UGT 1A4 (**A**). The contributions of these isozymes in this pathway were identified by recombinant UGT isozymes. Representative combined ultra-performance liquid chromatography quadrupole time-of-flight tandem mass spectrometry (UPLC-Q/TOF-MS) chromatograms of PPD and its glucuronidation metabolite; 0.5 mg/mL human liver microsomes (HLMs) or rat liver microsomes (RLMs) was incubated with PPD (100 μM) at 37 °C for 1 h in the presence of Uridine 5′-diphosphoglucuronic acid trisodium salt (UDPGA) (**B**).

A new metabolite designated as PPDG (glucuronidation metabolite of PPD) was eluted at 16.48 min by ultra-performance liquid chromatography quadrupole time-of-flight tandem mass spectrometry (UPLC-Q/TOF-MS) when PPD was incubated with pooled HLMs or RLMs in the presence of Uridine 5′-diphosphoglucuronic acid trisodium salt (UDPGA) (Figure 1B). The negative ion mode was used for structure identification because it is more sensitive than positive ion mode for PPD and its metabolite [15].

**Figure 2:**
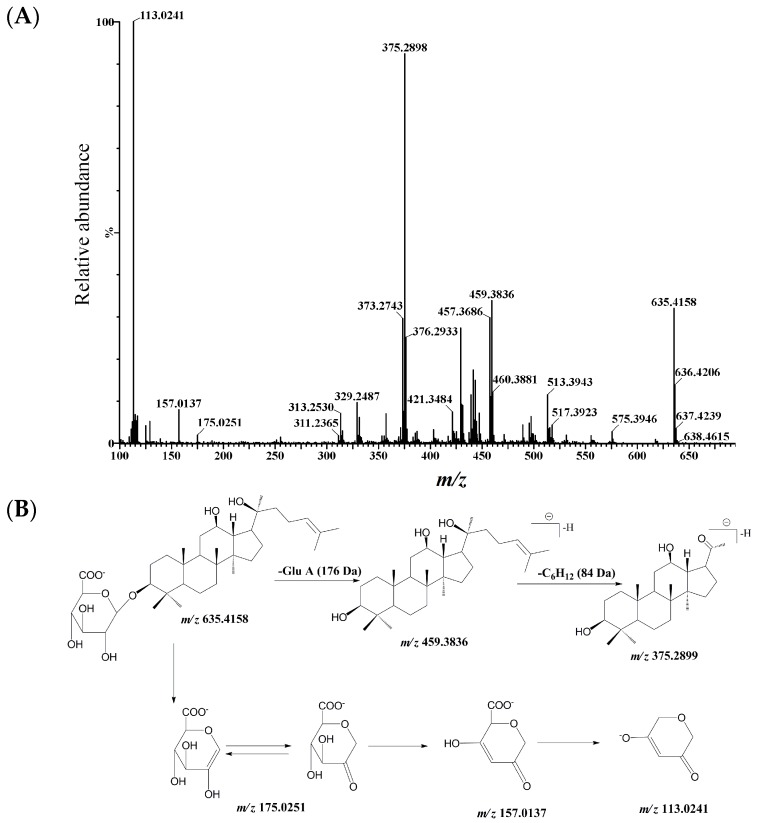
MS^2^ spectrum of PPDG (**A**) and its fragmentation pathways (**B**) in negative ion mode obtained from Q/TOF-MS. The MS^2^ data were obtained with *m*/*z* 635.41 ([M − H]^−^) as the precursor ion.

The Q/TOF-MS showed an exact deprotonated ion [M − H]^−^ at *m*/*z* 635.4158 (*Calcd.* 635.4159, elemental composition of C_36_H_59_O_9_) (Figure 2A), 176.0322 Da higher than that of PPD, suggesting conjugation with glucuronic acid. In the MS^2^ (product ions) spectrum, the typical neutral loss (−176.0322 Da) of a glucuronic acid moiety was observed from *m*/*z* 635.4158 to *m*/*z* 459.3836. The characteristic ion at *m*/*z* 375.2898 generated from the loss of –CH_2_CH_2_CH=C(CH_3_)_2_ at C-20 position (Figure 2B).

To confirm the substitution position of glucuronic acid moiety, standard of this metabolite was further biosynthesized by using pooled RLMs and structurally elucidated by high resolution mass and NMR analyses. In UPLC-Q/TOF-MS analysis, the biosynthesized product showed the identical retention time and MS^2^ spectrum to those of the microsome-generated product. The ^1^HNMR and ^13^CNMR data of PPDG were summarized in Table 1. Compared with that of PPD, the ^13^CNMR spectrum of PPDG showed a downfield shift of the C-3 signal (Δδ + 11.7 ppm) due to the glycosidation, while the signals at C-12 and C-20 remain unchanged, which indicated the glucuronic acid moiety substitution occurred at C-3 position. The anomeric configuration of the glucuronic acid was confirmed according to its coupling constant of the anomeric proton (δ 4.35 ppm, *J* = 7.8 Hz). Based on the evidence above, this metabolite was identified as PPD-3-*O*-β-d-glucuronic acid (Figure 1A).

**Table 1:** ^1^H and ^13^C NMR data of 20(*S*)-protopanaxadiol (PPD) and its glucuronidation metabolite (PPDG) (600 MHz, methanol-*d*_4_).

Position	^13^C NMR		^1^H NMR	
	PPD ^a^	PPDG	PPD ^a^	PPDG
1	39.4, t	39.0,t	-	-
2	28.2, t	29.4, t	-	-
3	78.4, d	90.1, d	-	-
4	39.5, s	38.6, s	-	-
5	56.4, d	56.1, d	-	-
6	18.8, d	17.8, d	-	-
7	35.2, t	34.6, t	-	-
8	40.0, d	39.6, d	-	-
9	50.5, d	50.0, d	-	-
10	37.4, s	36.6, s	-	-
11	32.1, t	31.7, t	-	-
12	71.0, d	71.9, d	3.9, m	4.1, m
13	48.6, d	48.2, d	-	-
14	51.7, s	51.2, s	-	-
15	31.4, t	30.7, t	-	-
16	26.8, t	26.0, t	-	-
17	54.8, d	53.7, d	2.33, *J* = 10.7, 7.1 ^b^	2.37, dd, *J* = 15, 7.8
18	15.9, q	15.4, q	0.99, s	1.06, s
19	16.4, q	15.7, q	0.87, s,	0.87, s
20	72.9, s	74.0, s	-	-
21	27.1, q	27.0, q	1.41, s	1.31, s
22	35.9, t	35.0, t	-	-
23	23.0, t	24.5, t	2.28, s	-
24	126.3, d	125.9, d	5.3, *J* = 7.1	5.1, dd, *J* = 7.6, 1.2
25	130.7, s	130.6, s	-	-
26	25.8, q	25.9, q	1.64, s	1.70, s
27	17.7, q	15.7, q	1.61, s	1.64, s
28	28.7, q	29.1, q	1.21, s	1.16, s
29	16.3, q	14.8, q	1.02, s	1.03, s
30	17.0, q	15.4, q	0.92, s	0.94, s
*Glucuronic acid*				
1′	-	106.4, d	-	4.35, d, *J =* 7.8
2′	-	76.3, d	-	-
3′	-	77.7, d	-	-
4′	-	73.5, d	-	-
5′	-	75.2, d	-	-
6′	-	175.3, s	-	-

^a^ Reporeted by [17]; ^b^ Coupling constant expressed as Hz.

### 2.2. Kinetics of 20(S)-Protopanaxadiol (PPD) Glucuronidation by Pooled Human Liver Microsomes (HLMs) and Rat Liver Microsomes (RLMs)

**Figure 3:**
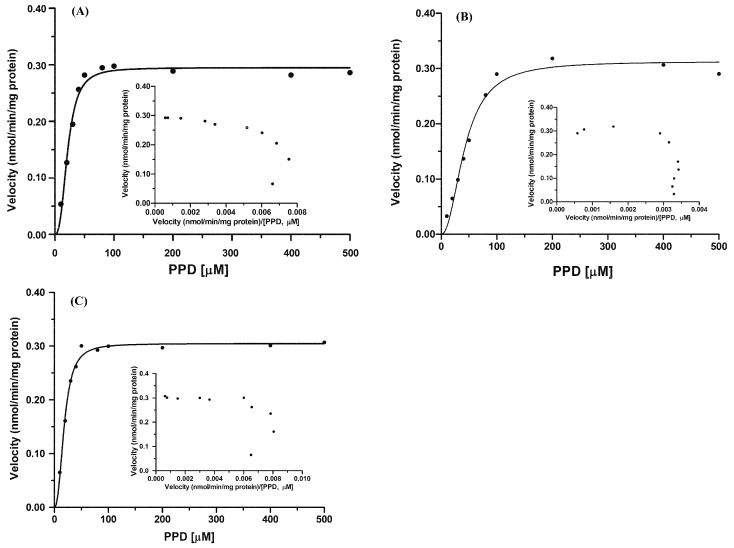
Enzyme kinetics of PPD glucuronidation by rat liver microsomes (RLMs) (**A**); human liver microsomes (HLMs) (**B**) and recombinant UGT1A4 (**C**). PPD (10–500 μM) was incubated with HLMs and RLMs (0.5 mg protein/mL) in the presence of UDPGA. An Eadie–Hofstee plot is shown as an inset. Data were obtained from three replicates.

Apparent enzyme kinetic parameters were evaluated by incubating PPD (10–500 μM) with pooled HLMs and RLMs. Sigmoidal kinetic model was best fitted to PPD glucuronidation by HLMs and RLMs (Hill model, Goodness of fit *R*^2^ > 0.97) with the Hill coefficient of >1 (positive cooperation), as evidenced by the Eadie-Hofstee plot (Figure 3). All the kinetic parameters are summarized in Table 2. The maximum formation rate of PPDG in the incubation containing HLMs was of 0.32 ± 0.01 nmol/min/mg protein, whereas the *S*_50_ (substrate concentration at 0.5 *V*_max_ (analogous to *K*_m_ in the Michaelis–Menten equation)) and *n* (Hill coefficient (which reflects the degree of sigmoidicity of the velocity versus substrate-concentration relationship)) of Hill component were of 42.80 ± 0.73 μM, and 2.12 ± 0.24, respectively. When PPD was incubated with RLMs, the maximum formation rate of PPDG was comparable with that in HLMs, whereas the *S*_50_ value of PPDG in RLMs was approximately 50% of that in HLMs. The maximum clearance in HLMs and RLMs were calculated to be 3.70 ± 0.01, 7.36 ± 0.27 μL/min/mg protein, respectively.

**Table 2:** Kinetic parameters for the formation of PPDG with pooled human liver microsomes (HLMs), rat liver microsomes (RLMs), and recombinant human UGT1A4 (Enzyme kinetic parameters were determined by Hill kinetics at 10–500 μM PPD. Data were expressed as mean ± SD).

Species	*V*_max_ (nmol/min/mg Protein)	*S*_50_ (μM)	*n*	*CL*_max_ (μL/min/mg Protein)	*R*^2^
RLMs	0.30 ± 0.01	20.68 ± 1.39	2.50 ± 0.48	7.36 ± 0.27	0.9793
HLMs	0.32 ± 0.01	42.80 ± 0.73	2.12 ± 0.24	3.70 ± 0.01	0.9753
UGT1A4	0.31 ± 0.01	18.49 ± 1.85	2.34 ± 0.23	8.39 ± 0.79	0.9894

### 2.3. Chemical Inhibition in Pooled HLMs

**Figure 4:**
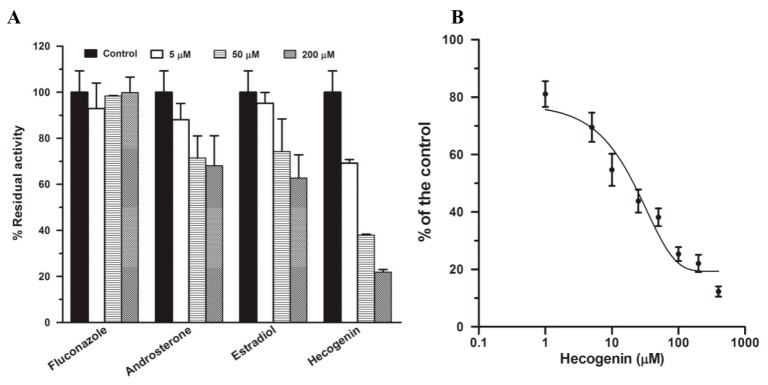
Chemical inhibition of PPD glucuronidation at a PPD concentration of 50 μM by four potent inhibitors (5, 50, and 200 μM) including fluconazole, hecogenin, estradiol, and androsterone in pooled HLMs (0.5 mg/mL) incubations. The incubations without inhibitors but the same volume of solvent were set as the control in which the activity of PPD glucuronidation was designated as 100% (**A**); Inhibitory effects of hecogenin on PPD glucuronidation. PPD glucuronidation was evaluated in pooled HLMs at the concentration of 50 μM in the presence of hecogenin (1–400 μM) (**B**). All data were obtained from three replicates.

Four potent chemical inhibitors including fluconazole, androsterone, estradiol, and hecogenin for UGT isozymes were used to inhibit PPD glucuronidation at concentrations of three levels (5, 50, and 200 μM). The result (Figure 4A) indicated that hecogenin showed concentration-dependent inhibitory effect on PPD glucuronidation by more than 60% compared with the solvent control at 50 µM; it is regared as most potent inhibiter on this biotransformation. The IC_50_ was determined to be 21.32 ± 4.31 μM (Figure 4B). Fluconazole, a selective inhibitor of UGT2B7, showed no inhibition on PPD glucuronidation, estradiol and androsterone showed weak inhibition of PPD glucuronidation. The results above indicated that UGT1A4 played a remarkable role in PPD glucuronidation.

### 2.4. Assay with Recombinant Human UGTs

**Figure 5:**
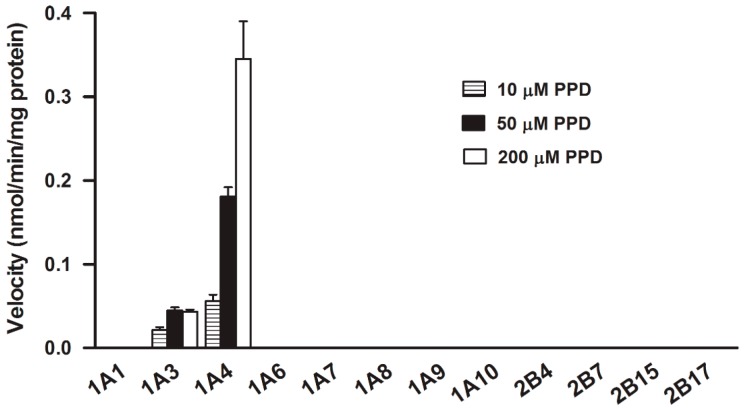
Assays of glucuronidation activities of PPD (10, 50, and 200 μM) catalyzed by different recombinant UGT isozymes (0.25 mg/mL). Data were obtained from three replicates.

To confirm the results obtained from the chemical inhibition study, PPD glucuronidation was assayed in twelve recombinant UGT isozymes, *i.e.*, UGT1A1, 1A3, 1A4, 1A6, 1A7, 1A8, 1A9,1A10, 2B4, 2B7, 2B15, and 2B17, at 0.25 mg protein/mL (Figure 5). With the PPD concentration of 10, 50, and 200 μM, UGT1A4 showed the highest and predominant catalytic activity (0.06 ± 0.01, 0.18 ± 0.01, and 0.35 ± 0.05 nmol/min/mg protein, respectively) towards to PPD glucuronidation. With the exception of UGT1A4, UGT1A3 also exhibited slight catalytic activity (0.02 ± 0.004, 0.04 ± 0.004, 0.04 ± 0.003 nmol/min/mg protein at 10, 50, and 200 μM, respectively). Other UGT isozymes tested did not participate in this metabolism.

### 2.5. Kinetics of PPD Glucuronidation by Recombinant UGT1A4

Because UGT1A4 showed the highest PPD glucuronidation activity, kinetic analysis for PPD glucuronidation in recombinant UGT1A4 was conducted using a wide range of concentrations (10–500 μM) of PPD. The PPDG formation by UGT1A4, as indicated by the Eadie–Hofstee plot (Figure 3C), exhibited Hill kinetics with the Hill coefficient of >1 (positive cooperation). The maximum formation rate was of 0.31 ± 0.01 nmol/min/mg protein, whereas the mean *S*_50_ was of 18.49 ± 1.85 μM, and the maximum clearance in recombinant UGT1A4 was of 8.39 ± 0.79 μL/min/mg protein.

## 3. Discussion

Ginsenosides and notoginsenosides were demonstrated to be the main bioactive components of *Panax notoginseng* and its corresponding preparations, which exhibited pharmacological activity against cardiovascular and cerebrovascular disorders [18,19,20]. Metabolism and pharmacokinetic study suggested that both PPD (protopanaxadiol) and PPT (protopanaxatriol)-type ginsenosides and notoginsenosides are susceptible to deglycosylation metabolism by gastric acid and/or intestinal bacteria *in vivo* [21,22]. PPD and PPT have been widely regarded as the final metabolites through the gastrointestinal tract, which may be responsible for the pharmacological activity of ginsenosides and notoginsenosides [1,2,3,4]. Currently, PPD is regarded as a potential antidepressant by early clinical trials [12]. However, information regarding the metabolic pathway of PPD after absorption from the digestive tract is very limited. *In vitro* phase I metabolism indicated that the predominant metabolic pathway of PPD was the oxidation of the double bond at Δ^(24,25)^ to yield 24,25-expoides, followed by hydrolysis and rearrangement to form the 24,25-vicinal diol derivatives and the 20,24-oxide form. PPD could not undergo direct glucuronidation which only occurred when it was transformed into 20,24-oxide form [2,14]. In our study, a glucuronidation metabolite of PPD (designated as PPDG) was first found and identified in pooled HLMs and RLMs using UPLC-Q/TOF-MS in negative ion mode. The chemical structure of PPDG was elucidated based on its accurate mass, mass spectral fragmentation patterns and NMR data. Our experiment suggested that hydroxyl group at C-3 position was susceptible to glucuronidation.

To identify and characterize the UGT isozymes that involved in PPD glucuronidation, optimization of incubation conditions in terms of linearity of metabolite formation with increased microsomal protein and incubation time were first performed with pooled HLMs. Subsequently, PPD glucuronidation activities in pooled HLMs and RLMs were determined. Then chemical inhibition study and a panel of twelve commercially available recombinant UGTs were used to identify the isozymes involved in PPD glucuronidation and their contributions. Finally, kinetics of PPD glucuronidation was performed in human recombinant UGT1A4. The kinetic profiles in pooled RLMs, HLMs and recombinant UGT1A4 followed the sigmoidal kinetic model (Hill profile), and the Hill coefficient was more than 1, which indicated that a positive cooperative reaction had occurred.

The selective inhibitors for UGT isozymes have not been identified as extensively as P450 isozymes [23]. To date, only two selective inhibitors have been identified: hecogenin for UGT1A4 [24] and fluconazole for UGT2B7 [25], which were applied in the present study and hecogenin showed the most significant inhibitory effect with the IC_50_ of 21.32 ± 4.31 μM (Figure 4), suggesting that UGT1A4 was the most important isozyme catalyzing the PPD glucuronidation. Additionally, androsterone and estradiol, as other two potent inhibitors, were also used in this study. It had been demonstrated that androsterone is a potent inhibitor for UGT1A9 and UGT2B7; however, it showed only slight inhibition even at a high concentration, suggesting that UGT1A9 and UGT2B7 were not involved in PPD glucuronidation. It has been reported that androsterone also inhibited UGT1A4 slightly [25], which may be the reason for its slight inhibition of PPD glucuronidation. The phenolic glucuronidation of β-estradiol at C-3 position is mainly catalyzed by UGT1A1 with atypical kinetics [26,27], whereas the alcoholic glucuronidation of β-estradiol at C-17 position is mostly catalyzed by UGT2B7 [28]. However, studies indicate UGT1A8, 1A10, and 1A3 can also catalyze the glucuronidation of β-estradiol at C-3 position with significant activities [29], whereas UGT1A3, 1A4, 1A8, 1A10, and 2B7 may also take part in the glucuronidation of β-estradiol at C-17 [29]. In this experiment, estradiol was used as an inhibitor to inhibit PPD glucuronidation. As shown in Figure 4, estradiol showed slight inhibitory effect on PPD glucuronidation, suggesting that UGT1A1, UGT1A3, UGT1A8 and UGT2B7 were not involved in PPD glucuronidation. The slight inhibition may be attributed to the slight inhibition of UGT1A4. All the chemical inhibition study indicated that UGT1A4 was the predominant isozyme catalyzing PPD glucuronidation. The conclusion reached was confirmed by twelve recombinant UGT isozymes analyses. As shown in Figure 5, UGT1A4 showed the highest and predominant catalytic activity. Besides UGT1A4, UGT1A3 also catalyze this metabolism, but the efficiency was rather low. Other isozymes tested did not catalyze this reaction.

Human UGT known to metabolize xenobiotics are the products of two gene families, UGT1 and UGT2,and human hepatic UGT enzymes include UGT 1A1, 1A3, 1A4, 1A6, 1A9, 2B4, 2B7, 2B10, 2B11, 2B15 and 2B17 [16]. Although other isozymes that were not tested in the present study may participate in PPD glucuronidation, it could be concluded that UGT 1A4 was the major isozyme involved in PPD glucuronidation according to chemical inhibition and recombinant isozyme analyses, which was further confirmed by the enzymatic parameters in pooled HLMs that were comparable to those in recombinant UGT 1A4.

## 4. Experimental Section

### 4.1. Materials

PPD and hecogenin were obtained from Shanghai PureOne Biotechnology (purity > 98%; Shanghai, China). Ginsenoside Rg1 (GRg1) was provided by the Shanghai R&D Center for Standardization of Traditional Chinese Medicine (purity > 98%; Shanghai, China). Uridine 5′-diphosphoglucuronic acid trisodium salt (UDPGA) was purchased from Santa Cruz Biotechnology, Inc. (Santa Cruz, CA, USA). Alamethicin, Tris base, estradiol, fluconazole, and magnesium chloride were purchased from Sigma-Aldrich (St. Louis, MO, USA). Androsterone was obtained from Dr. Ehrenstorfer GmbH (Augsburg, Germany). Pooled human liver microsomes (HLMs, prepared from livers of 20 human donors) and human recombinant UGT 1A1, 1A3, 1A4, 1A6, 1A7, 1A8, 1A9, 1A10, 2B4, 2B7, 2B15, and 2B17 were purchased from BD Gentest™ (Woburn, MA, USA). Pooled rat liver microsomes (RLMs) were prepared according to the methods described as previous report [30] in our laboratory and protein concentration was determined by Bradford assay. The protein concentration was adjusted to 10 mg/mL using the Tris–HCl (50 mM, pH 7.4) buffer, and then 1 mL aliquots were dispensed into labeled tubes and stored at −80 °C. All other chemicals and reagents were of analytical grade and commercially available. Experiment related to animals was performed on the basis of the National Institute of Health Guidelines on the principles of animal care (2004) and approved by the Institutional Animal Care and Use Committee, Shanghai University of Traditional Chinese Medicine (Shanghai, China; 1 November 2010).

### 4.2. Glucuronidation of PPD with the Pooled HLMs and RLMs

*In vitro* PPD glucuronidation was in an incubation volume of 100 μL. Incubation conditions were initially optimized for the linear product formation with respect to protein concentration (0.2–1 mg/mL), and incubation time (15–120 min). The stock solution of PPD was prepared in dimethyl sulfoxide (DMSO), and the final concentration of DMSO in the incubations was 1% (*v*/*v*). Briefly, the incubation mixture contained HLMs or RLMs (0.5 mg of protein/mL), UDPGA (2 mM), MgCl_2_ (4 mM), alamethicin (25 μg/mg protein), PPD (10–500 μM), and Tris–HCl buffer (50 mM, pH 7.4). The mixture was incubated for 5 min at 37 °C, and then the reaction was started by the addition of UDPGA and incubated in a shaking water bath for 60 min. The reaction was terminated by addition of 200 μL of ice-cold acetonitrile containing GRg1 (internal standard (IS), 0.5 μg/mL) and then centrifuged at 15,000× *g* for 10 min at 4 °C. The supernatant was evaporated to dryness under nitrogen gas at room temperature, and the residue was reconstituted with 200 µL of 20% acetonitrile. After centrifugation at 15,000× *g* for 10 min at 4 °C, 5 μL of the supernatants was subjected to analysis. Incubations without PPD and UDPGA were served as blank and negative controls, respectively.

### 4.3. Identification of the Metabolite by UPLC-Q/TOF-MS

Identification of metabolite was carried out on ultra-performance liquid chromatography quadrupole time-of-flight tandem mass spectrometry (UPLC-Q/TOF-MS) (Waters Corporation, Milford, MA, USA) as described in detail previously [15,31]. The peak at RT (retention time) 19.30 min was detected as PPD by Q/TOF-MS analysis (Figure 1B).

### 4.4. Preparation and Structural Elucidation of Metabolite

The glucuronidation metabolite of PPD (PPDG) was prepared by biotransformation and purified by preparative HPLC system (Waters Corporation, Milford, MA, USA) for structural elucidation and quantitative analysis. Enzymatic biotransformation of the target metabolite was conducted using pooled RLMs. In brief, 500 μM PPD was incubated with pooled RLMs (1 mg of protein/mL), 50 mM Tris–HCl (pH 7.4), 4 mM MgCl_2_, Brij 58 (0.5 mg/mg protein), and 2 mM UDPGA in 100 mL final incubations for 2 h at 37 °C. The stock solution of PPD (50 mM) was prepared in DMSO. The concentration of organic solvent in the final incubation was 1%. The reaction was terminated by addition of 200 mL of ice-cold acetonitrile. After removal of protein by centrifugation at 15,000× *g* for 10 min at 4 °C, the supernatant was evaporated to dryness *in vacuo*.

The residue was dissolved in 10 mL of 80% methanol and then subjected to a Waters LC-MS controlled preparative HPLC Auto Purification System (Milford, MA, USA). The system was comprised of a 2767 Sample Manager, a 2545 binary high-pressure LC Pump, a column/fluidic organizer (SFO), a 515 make up pump, a 2489 UV detector, a ZQ single-quadrupole mass spectrometer (Waters Corporation) equipped with a Z-spray electrospray source. The SFO contains an accurate splitter (1:10,000). The complete system was controlled by MassLynx^TM^ software version 4.1 (Waters Corporation). The mobile phase consisted of acetonitrile and water with a linear gradient from initially 10% to 90% acetonitrile over 10 min. The flow rate was set at 15 mL/min. The target metabolite was eluted at 6.5 min, and lyophilized to get a white powder.

The structure of metabolite was identified by ^1^HNMR and ^13^CNMR spectroscopies. All the experiments were recorded on a Bruker AV 600 NMR spectrometer (Bruker, Newark, Germany). The purified metabolite was stored at −20 °C before dissolving in methanol-*d*_4_ for NMR analysis. Chemical shifts were given on a δ scale and referenced to Tetramethylsilane (TMS) at 0 ppm for ^1^H NMR (600 MHz) and ^13^C NMR (150 MHz).

### 4.5. Quantitative Analysis by LC-MS/MS

Quantitative analysis was conducted on an Agilent 1290 infinity system (Agilent Technologies, Santa Clara, CA, USA) coupled with an MS/MS detector (6410 B Triple Quad LC/MS; Agilent Technologies) in negative ion mode. The chromatographic separations were performed on an ACQUITY UPLC HSS T_3_ column (100 mm × 2.1 mm i. d. (inner diameter), 1.8 μm) thermostated at 45 °C. The mobile phase consisted of A (5 mM ammonium acetate in water) and B (acetonitrile) at a flow rate of 0.4 mL/min. The gradient elution program was set as follow: 0–2 min 20%–50% B, 2–4 min 50%–60% B, 4–5 min 60% B and finally, reconditioning the column with 20% B for 1 min. Optimized ion source parameters were set as follows: gas temperature 325 °C; gas flow 10 L/min; nebulizer 35 psi; capillary voltage 3500 V; fragmentor voltage 265 V for PPDG and 215 V for IS; cell accelerator voltage 0 V; dwell time 200 ms. Data were acquired in multiple reaction monitor (MRM) mode for the following transitions: *m*/*z* 635.4 to *m*/*z* 375.1 for PPDG and *m*/*z* 799.5 to *m*/*z* 637.6 for IS, with the collision energy being set at 50 and 26 V, respectively.

### 4.6. Chemical Inhibition in Pooled HLMs

Fluconazole [24,25], androsterone [25,32], estradiol [26,33], and hecogenin [25,34] were used to inhibit their potential inhibitory effects on PPD glucuronidation in pooled HLMs. A set of concentrations (5, 50, and 200 μM) was used for all four inhibitors. The concentration of PPD was set at 50 μM. The reaction time and protein concentration were set at 60 min and 0.5 mg/mL, respectively. All other incubation conditions were the same as those for Glucuronidation of PPD in pooled HLMs described above. The concentration of organic solvent was 1% of the total incubation volume. An experimental group without inhibitors but with solvents was used as the control, in which the activities of PPD glucuronidation were designated as 100%. The activities of PPD glucuronidation in the inhibited samples were compared with the control to evaluate the remaining enzyme activity. For determination of IC_50_ (representing the concentration that inhibits 50% of the control activity) of hecogenin, experiments with a set of concentrations of hecogenin (1–400 μM) in pooled HLMs were conducted.

### 4.7. Incubation with Recombinant Human UGTs

PPD glucuronidation was measured in reaction mixtures containing human recombinant UGT1A1, 1A3, 1A4, 1A6, 1A7, 1A8, 1A9, 1A10, 2B4, 2B7, 2B15, and 2B17. Three substrate concentrations (10, 50, and 200 μM) were used in this study. The incubation conditions were the same as those for Glucuronidation of PPD in pooled HLMs described above except for the protein concentration (0.25 mg/mL).

### 4.8. Kinetics of PPD Glucuronidation in Recombinant UGT 1A4

A kinetic study for PPD glucuronidation by human recombinant UGT 1A4 was also conducted by incubating PPD with human recombinant UGT 1A4 at protein concentration of 0.25 mg/mL for 1 h. All other incubation conditions were identical to those of glucuronidation of PPD in pooled HLMs.

### 4.9. Enzyme Kinetic Data Analysis

All results were obtained from three replicates in different microsomal incubations, and all the data were expressed as mean ± SD. To estimate the kinetic parameters, data were transformed and Eadie–Hofstee curves were plotted, which help to identify kinetic models. Then kinetic parameters were obtained by fitting velocity data to the sigmoidal kinetic (Hill) model (Equation (1)) using Graph Pad Prism 5.0 software (San Diego, CA, USA). In addition, *CL*_max_ was calculated according to the Equation (2).

(1) $$V=\frac{V_{\max}\;\times \;{\left[S\right]}^{n}}{S_{50}^{n}\;+\;{\left[S\right]}^{n}}$$

(2) $$CL_{\max}\;=\;\frac{V_{\max}}{S_{50}}\;\times \;\frac{n-1}{n{\left(n-1\right)}^{1/n}}$$

*V* is reaction velocity, *V*_max_ is the maximum reaction velocity, [*S*] is the substrate concentration, *S*_50_ is the substrate concentration at 0.5 *V*_max_ (analogous to Km in the Michaelis–Menten equation), *n* is the Hill coefficient (which reflects the degree of sigmoidicity of the velocity *versus* substrate-concentration relationship), *CL*_max_ is the maximum clearance, which provides an estimate of the highest clearance attained.

## 5. Conclusions

In our present study, the glucuronidation of PPD in pooled RLMs and HLMs was first investigated and the metabolite was identified as PPD-3-*O*-*β*-d-glucuronide by comparison with a biosynthesized product of which the structure was identified via UPLC-Q/TOF-MS and NMR analyses. The major isozyme involved in PPD glucuronidation was UGT1A4. In addition, UGT1A3 also showed a very slight effect. More studies on the biological activities of this glucuronidation metabolite are needed.
